# Quantum Dots-Based Immunofluorescent Imaging of Stromal Fibroblasts Caveolin-1 and Light Chain 3B Expression and Identification of Their Clinical Significance in Human Gastric Cancer

**DOI:** 10.3390/ijms131113764

**Published:** 2012-10-24

**Authors:** Yuyu He, Xianda Zhao, Jun Gao, Lifang Fan, Guifang Yang, William Chi-shing Cho, Honglei Chen

**Affiliations:** 1 Department of Pathology, School of Basic Medical Science, Wuhan University, Wuhan 430071, China; E-Mails: yuyuhe@whu.edu.cn (Y.H.); xiandazhao.md@whu.edu.cn (X.Z.); lifangfan@whu.edu.cn (L.F.); 2 Department of Molecular Pathology, Wuhan Nano Tumor Diagnosis Engineering Research Center, Wuhan 430075, China; E-Mail: gaojunemail@126.com; 3 Department of Pathology, Zhongnan Hospital of Wuhan University, Wuhan 430071, China; E-Mail: guifangyang@yahoo.com.cn; 4 Department of Clinical Oncology, Queen Elizabeth Hospital, 30 Gascoigne Road, Kowloon, Hong Kong; 5 Department of Biochemistry, Rush University Medical Center, 1735 West Harrison Street, Chicago, IL 60612, USA

**Keywords:** autophagy, cancer-associated fibroblast, caveolin-1, gastric cancer, light chain 3B, quantum dots, tumor microenvironment

## Abstract

Caveolin-1 (Cav-1) expression deficiency and autophagy in tumor stromal fibroblasts (hereafter fibroblasts) are involved in tumor proliferation and progression, particularly in breast and prostate cancer. The aim of this study was to detect the expression of fibroblastic Cav-1 and LC3B, markers of autophagy, in gastric cancer (GC) and to analyze their clinical significances. Furthermore, because Epstein-Barr virus (EBV)-associated GC (EBVaGC) is a unique subtype of GC; we compared the differential expression of fibroblastic Cav-1 and LC3B in EBVaGC and non-EBVaGC. Quantum dots (QDs)-based immunofluorescence histochemistry was used to examine the expression of fibroblastic Cav-1 and LC3B in 118 cases of GC with adequate stroma. QDs-based double immunofluorescence labeling was performed to detect the coexpression of Cav-1 and LC3B proteins. EBV-encoded small RNA was detected by QDs-based fluorescence *in situ* hybridization to identify EBVaGC. Multivariate analysis indicated that low fibroblastic Cav-1 level was an independent prognosticator (*p* = 0.029) that predicted poorer survival of GC patients. Positive fibroblastic LC3B was correlated with lower invasion (*p* = 0.032) and was positively associated with Cav-1 expression (*r* = 0.432, *p* < 0.001). EBV infection did not affect fibroblastic Cav-1 and LC3B expression. In conclusion, positive fibroblastic LC3B correlates with lower invasion, and low expression of fibroblastic Cav-1 is a novel predictor of poor GC prognosis.

## 1. Introduction

Tumor stroma is increasingly recognized as an active participant in tumor progression, metastasis and drug resistance [1,2]. Although the complex mechanisms of these processes need to be further dissected, the altered expression of stromal proteins have been manifested as novel biomarkers in various types of human cancers, including breast [3–8], prostate [9], nasopharynx [10], basal cell cancers [11] and melanoma [12]. Gastric cancer (GC) is the fourth most commonly occurring cancer and the third most lethal cancer worldwide [13], it is more prevalent in men than women in China [14]. However, the clinical significance of GC stromal proteins has been largely unexplored. Therefore, it is worthwhile to elucidate the clinical significance of specific stromal proteins that are involved in GC progression.

Caveolin-1 (Cav-1) is a multifunctional scaffolding protein with multiple binding partners that are associated with cell surface caveolae and the regulation of lipid raft domains. It is well known that Cav-1 regulates multiple cancer-associated processes in cancer cells, including cell proliferation, migration and metastasis, cell death and survival, and multidrug resistance [15]. Cav-1 influences tumor progression not only in cancer cells but also in tumor stroma [1]. Studies of breast cancer have revealed that the loss of Cav-1 in cancer-associated fibroblast cells (CAFs) promotes breast cancer proliferation and progression by remodeling the tumor microenvironment, protecting cancer cells from apoptosis and other mechanisms [1,16,17]. Survival analysis indicates that the absence of fibroblastic Cav-1 expression is a powerful independent predictor of early disease recurrence, metastasis and adverse outcome in breast cancer [3,4,6], confirming its tumor-promoting role.

Autophagy is a cellular homeostatic mechanism that involves protein and organelle degradation and has a number of connections to either tumor promotion or inhibition. *In vitro* studies using a coculture system of the breast cancer cell line MCF7 and fibroblasts have demonstrated that activated autophagy in fibroblasts is the primary cause of fibroblastic Cav-1 degradation [1,16,18]. Furthermore, autophagy also promotes tumor development synergistically with Cav-1 degradation through the metabolic/catabolic reprogramming of CAFs to fuel the growth of adjacent tumor cells [1,16,19–21]. Microtubule-associated protein light chain 3B (LC3B) localizes to the autophagosome membrane and is therefore widely used as a marker of autophagy [22]. Hence, LC3B expression in GC fibroblasts was also evaluated in our investigation.

Fluorescent semiconductor nanocrystal quantum dots (QDs) are a novel class of multifunctional inorganic fluorophores that have promising utility in biological imaging [23–26]. The beneficial properties of QDs compared to organic fluorophores are narrow emission band peaks, broad absorption spectra, intense signals and remarkable resistance to photobleaching [26]. Moreover, the optical properties of QDs, in particular the wavelength of their fluorescence, depend strongly on their sizes [27]. Different QD colors can be simultaneously excited by a single light source with minimal spectral overlapping. These properties make QDs extremely useful for multiplexed molecular immunofluorescent imaging, which is an advanced technique for studying the clinicopathological characteristics of molecular subtypes and tumor prognosis.

Based on the above information, we hypothesized that low fibroblastic Cav-1 levels and high autophagy levels may promote GC development. Using the established QDs-based immunofluorescence histochemistry (QDs-IHC) and QDs-based double immunofluorescent labelling methods, we focused on the expression of fibroblastic Cav-1 and LC3B in GC, followed by analysis of the correlation with GC prognosis. Because Epstein-Barr virus (EBV)-associated gastric cancer (EBVaGC) is a unique subtype of GC and has features as the monoclonal proliferation of EBV-infected epithelial cells [28,29], we also detected EBV-encoded small RNA (EBER) via QDs-based fluorescence *in situ* hybridization (QDs-FISH) to investigate the influence of EBV infection on fibroblastic Cav-1 and LC3B expression.

## 2. Results and Discussion

### 2.1. Expression of Cav-1 and LC3B in GC

**Figure 1:**
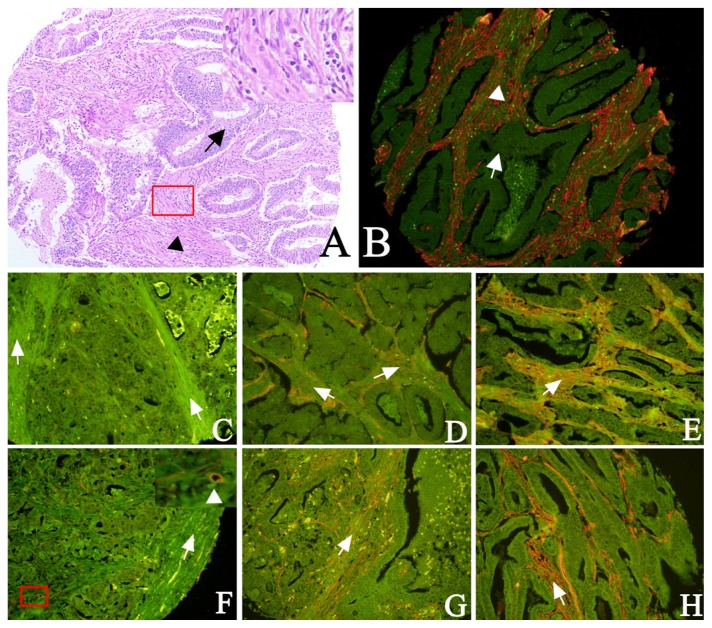
Identification of fibroblasts by H and E staining and detection of Cav-1 and LC3B proteins by QDs-IHC. **A**, **B**: arrows indicate tumor cells and triangles indicate fibroblasts. **C**–**E**: fibroblastic LC3B staining intensity was scored as 0 (negative, **C**), 1 (weak, **D**), or 2 (strong, **E**). **F**–**H**: fibroblastic Cav-1 staining intensity was scored as 0 (negative, **F**), 1 (weak, **G**), or 2 (strong, **H**). The enlarged region of F showed endothelial cells in the blood vessel used as a positive internal control (**A**, **B**: 100× magnification; **C**–**H**: 200× magnification; boxed region in **A** and **F** are enlarged in the upper right corner of panels **A** and **F**).

**Figure 2:**
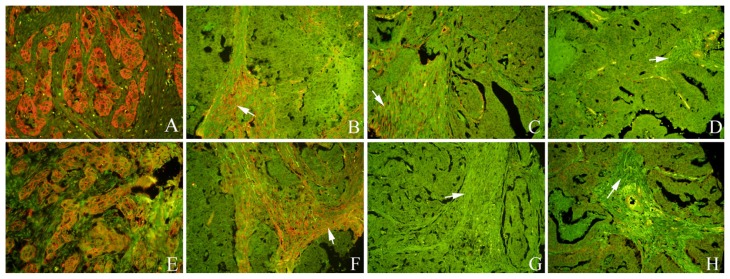
QDs-IHC-based localization of Cav-1 and LC3B in tumor cells and staining patterns. **A**, **E**: Cav-1 and LC3B located at the tumor cell membrane. **B**, **F**: Cav-1 and LC3B-positive fibroblasts; **C**, **G**: Cav-1-positive and LC3B-negative fibroblasts; **D**, **H**: Cav-1- and LC3B-negative fibroblasts. (White arrows indicates stroma; **A**–**D**: Cav-1 staining; **E**–**H**: LC3B staining; **A**, **E**: 400× magnification; **B**–**D** and **F**–**H**: 200× magnification; **B** and **F**, **C** and **G**, **D** and **H**: serial sections).

We detected Cav-1 and LC3B protein expression in epithelial and stromal compartments via QDs-IHC. One set of tissue microarrays (TMAs) was used for hematoxylin and eosin (H and E) staining to identify and ensure the differential detection and evaluation of the tumor cells and fibroblasts (Figure 1A,B). Serial sections were used for H and E staining and QDs-IHC. The different staining intensities of fibroblastic Cav-1 and LC3B are illustrated in Figure 1. In the epithelial region, Cav-1 and LC3B immunoreactivity was predominately located at the tumor cell membrane (Figure 2A,E). Representative expression patterns of Cav-1 and LC3B in fibroblasts from serial sections are shown in Figure 2.

### 2.2. Clinical Significance and Prognostic Value of Fibroblastic Cav-1 and LC3B

To investigate the effect of fibroblastic Cav-1 and LC3B expression on tumor aggressiveness, we examined the relationship between fibroblastic Cav-1 and LC3B expression and clinicopathological features including age, gender and differentiation grade. However, as shown in Table 1, we did not find any significant association between fibroblastic Cav-1 levels and the various clinicopathological features. Additionally, fibroblastic LC3B was not significantly associated with any clinicopathological features except the depth of invasion (*p* = 0.032, Table 1).

**Figure 3:**
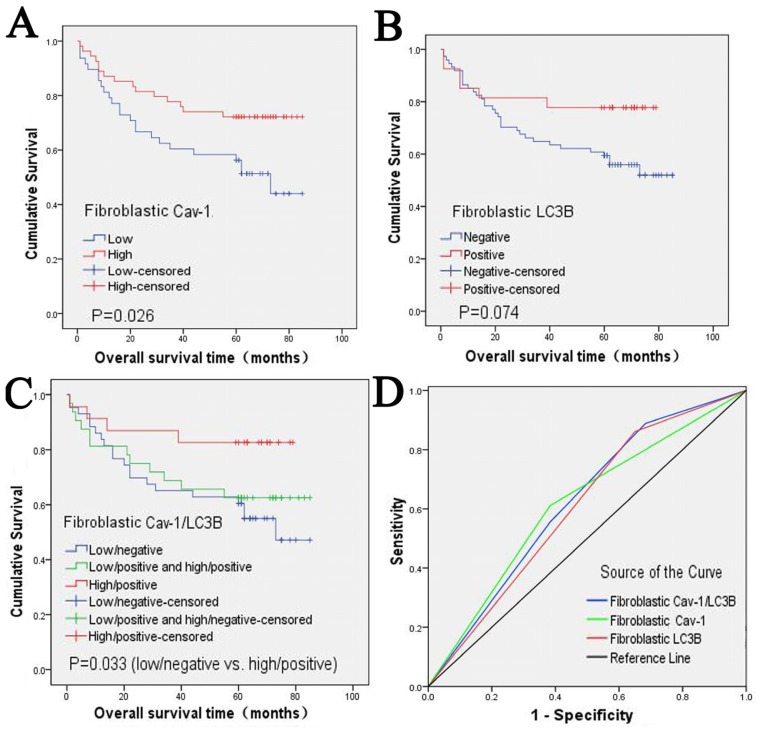
Cumulative survival curves of GC patients and Receiver Operating Characteristic analysis. **A**: The low fibroblastic Cav-1 group was correlated with poorer survival of GC patients. **B**: Although the high fibroblastic LC3B group showed a better survival trend, it was not statistically significant. **C**: The combined features of fibroblastic Cav-1 and LC3B also significantly predicted prognosis. **D**: The combined fibroblastic Cav-1 and LC3B status had the largest area under the curve compared with the fibroblastic Cav-1 level and LC3B level.

To explore the prognostic values of fibroblastic Cav-1 and LC3B, we used the Kaplan-Meier method to estimate the survival curve of overall survival and the log-rank test to assess the difference in survival between the fibroblastic Cav-1 and LC3B expression subgroups. The cumulative five-year survival rate of patients whose tumors exhibited high fibroblastic Cav-1 expression was 72.2% (95% CI: 60.2%–84.2%), whereas it was only 56.2% (95% CI: 42.1%–70.3%) in the low fibroblastic Cav-1 group. The median overall duration of survival of the low fibroblastic Cav-1 expression group was 73.0 months (95% CI: 53.725–92.275). After a full follow-up period, the high stromal Cav-1 expression group had a calculated survival rate of approximately 75% (Figure 3A). As shown in the survival curve, the high fibroblastic Cav-1 expression group had a better survival rate and the difference between the fibroblastic Cav-1 subgroups was statistically significant (*p* = 0.026, Figure 3A). Although the high fibroblastic LC3B expression group had a better survival rate, no significant difference in overall survival was found between the fibroblastic LC3B levels (*p* = 0.074, Figure 3B). When we combined the expression status of fibroblastic Cav-1 and LC3B, the difference of overall survival between Cav-1(−)/LC3B(−) and Cav-1(+)/LC3B(+) was significant (*p* = 0.033, Figure 3C).

In addition, we used the COX proportional hazard regression model to investigate the independent predictors of GC. In univariate analyses, the classic clinicopathological features such as TNM stage, T classification and lymph node, were found to be significantly associated with the overall survival of GC patients (*p* = 0.003, 0.001 and 0.034, respectively, Table 2). The fibroblastic Cav-1 level and LC3B level was also associated with overall survival. Compared to low/negative fibroblastic Cav-1/LC3B expression, high/positive Cav-1/LC3B expression significantly diminished the risk of death in GC patients (*p* = 0.021, 0.041, respectively, Table 2). The factors that were significant according to univariate analysis were subjected to multivariate analysis. Multivariate analysis revealed that only fibroblastic Cav-1 and TNM stage were independent prognosticators (*p* = 0.029 and 0.001, respectively); fibroblastic LC3B and other clinicopathological features failed to independently predict GC prognosis (Table 2).

The fibroblastic Cav-1 and LC3B levels and their combined status were further studied by receiver operating characteristic (ROC) analysis to assess their predictive value for death. As showed in Figure 3D, the combined status of fibroblastic Cav-1 and LC3B predicted death with good performance (*p* < 0.05); the area under the curve was 0.622 (95% CI: 0.509–0.735). However, the fibroblastic Cav-1 and LC3B levels failed to predict death, with areas under the curve of 0.614 (95% CI: 0.497–0.731) and 0.606 (95% CI: 0.492–0.719), respectively (both *p >* 0.05) (Figure 3D).

### 2.3. The Correlation between Fibroblastic Cav-1 and LC3B

**Figure 4:**
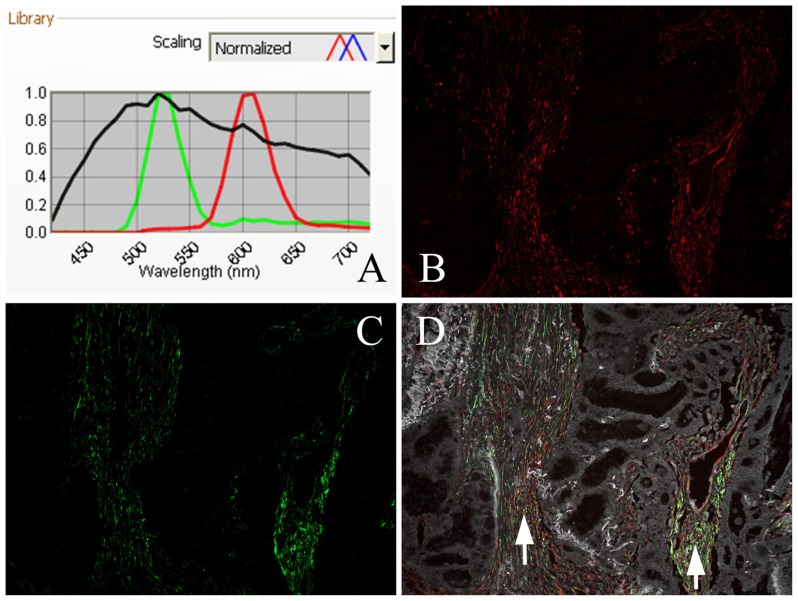
Coexpression of Cav-1 and LC3B proteins detected by QDs-based double immunofluorescent labeling in GC tissue and analyzed by a multispectral imaging system. **A**: Emission spectra of QDs (545 nm-green; 605 nm-red) and tissue autofluorescence (black); **B**: Bright red, Cav-1-positive signal; **C**: Green, LC3B-positive signal; **D**: Distinct coexpression of Cav-1 and LC3B proteins; fibroblasts are positive (white arrow), tubular adenocarcinoma is negative. (**B**–**D**: 200× magnification).

The results of QDs-IHC were used to investigate the correlation between fibroblastic Cav-1 and LC3B. As shown in Table 1, among the 45 cases with low fibroblastic Cav-1expression, 42 (93.3%) cases showed negative expression of LC3B, and among the 51 cases with high fibroblastic Cav-1 expression, 23 (45.1%) cases were LC3B-positive and manifested a positive correlation (*r* = 0.432, *p* = 0.000). To visualize the relationship between the expression of fibroblastic Cav-1 and LC3B, QDs-based double immunofluorescence labeling was conducted. As shown in Figure 4, we used a multispectral microscopy imaging system to assay the different spectra of QDs. The positive signal of Cav-1 was bright red and the LC3B signal was green. The coexpression of Cav-1 and LC3B proteins can be observed in Figure 4D.

### 2.4. EBV Infection Affected Expression of Fibroblastic Cav-1 and LC3B

**Figure 5:**
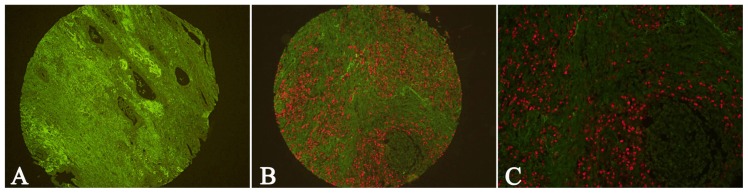
Detection of EBER by QDs-FISH in GC tissues. **A**: Negative EBER signal in GC tissue; **B**, **C**: Positive EBER signal in GC tissue. (**A**, **B**: 100× magnification; **C**: 200× magnification).

The EBER signal was detected by QDs-FISH with biotin-labeled RNA probes. In GC tissue, the EBER-positive signal was located in the nuclei of tumor cells (Figure 5B,C). Figure 5A shows EBV-negative GC tissue. QDs-FISH reactions for EBER were positive in 17.79% (21/118) of the GC tissues. In addition, we matched the results of EBER, fibroblastic Cav-1 and LC3B; however, no significant correlation among EBV infection, fibroblastic Cav-1 and LC3B was demonstrated (Table 3).

### 2.5. Discussion

In our study, the novel fluorescent semiconductor nanocrystals QDs successfully detected the expression and clinical significance of Cav-1 and LC3B in the fibroblasts of GC tissue. Additionally, we developed a method for the simultaneous imaging of Cav-1 and LC3B from which distinct positive signals for each protein were obtained. The coexpression of Cav-1 and LC3B in fibroblasts was also accurately demonstrated with high resolution. Compared with conventional immunohistological staining, QDs-based multiplexed molecular imaging provides a holistic approach to vividly observing the heterogeneous expression of proteins in different cells.

In recent years, the prognostic value of fibroblastic Cav-1 has been widely confirmed in breast cancer [3,4,6], and the tumor-promoting role of low fibroblastic Cav-1 expression has been manifested. On one hand, Cav-1 interacts with the activin receptor-like kinase (ALK) 1, a type I TGF-β receptor (TβR I), and suppresses the TGF-β-mediated phosphorylation of Smad-2 and subsequent downstream events [30,31]. In tumor stroma, loss of fibroblastic Cav-1 induces the aberrant activation of the TGF-β pathway, consequently transforming the fibroblasts to activated CAFs, which play important roles in fibrosis and extracellular matrix remodeling [1,7,8,16,32]. On the other hand, the loss of fibroblastic Cav-1 promotes tumor progression by promoting the metabolic/catabolic reprogramming of CAFs synergistically with autophagy to fuel the growth of adjacent tumor cells [1,7,16–18]. In our study, we assessed the clinical significance of fibroblastic Cav-1 in GC and found that the fibroblastic Cav-1 level showed no correlation with classic clinicopathological features (Table 1) but predicted a short survival of GC patients (*p* = 0.026). Our multivariate analysis further identified fibroblastic Cav-1 as a new independent prognostic marker for GC (*p* = 0.029). Clearly, our data were consistent with our hypothesis that low expression of fibroblastic Cav-1 can predict poor survival in GC, as previously demonstrated in breast cancer [3,4,6].

ROC curve analysis enabled the determination of the parameters that have predictive values of death. We have conducted ROC curve analysis of the fibroblastic Cav-1 level, LC3B level and their combined status. We found that the fibroblastic Cav-1 level and LC3B level could not predict death individually, but the combined detection of fibroblastic Cav-1 and LC3B showed significant predictive value, which suggests that they may be regarded as correlative predictive factors of death for GC patients. Combining the results of Kaplan-Meier analysis, log-rank test and multivariate analysis, we can conclude that fibroblastic Cav-1 is an independent prognostic factor of the overall survival and risk of death; moreover, fibroblastic Cav-1 combined with LC3B predicts the death of GC patients with improved accuracy. Although stromal Cav-1 status can be used as a prognostic predictor, when the correlation between stromal Cav-1 levels and the classic clinicopathological parameters of GC was analyzed, no significant association was found. In fact, all studies in breast cancer indicate that the loss of stromal Cav-1 correlates with poor survival but not always with adverse clinicopathological parameters [3,4,6,7,33–36]. Nevertheless, the loss of stromal Cav-1 has great predictive value in ER(+), PR(+), HER2(+) and triple-negative patients (ER(−)/PR(−)/HER2(−)). Moreover, endocrine therapy, such as tamoxifen in ER(+) patients, does not influence its predictive value. This makes stromal Cav-1 a new “universal” or “widely applicable” breast cancer prognostic marker [33]; however, a study in GC with larger sample size is needed to further confirm the correlation between stromal Cav-1 and clinicopathological parameters. Additionally, prospective clinical trials need be conducted to validate the prognostic value of stromal Cav-1 in breast cancer and GC to determine whether stromal Cav-1 status can be used as an independent classification system for cancer therapy and whether it merits further study.

Currently, the main role of autophagy is to act as an adaptation to metabolic stress, such as starvation, hypoxia and oxidative stress. In some situations, autophagy is also regarded as a form of “autophagic programmed cell death” [16,37,38]. With continuous growth of tumors, the tumor microenvironment becomes sensitive to autophagy. Autophagy occurs in tumor cells and stromal cells, in which autophagy leads to Cav-1 degradation [1,16,19,39]. Therefore, we also assessed the clinical significance of LC3B in the fibroblasts of GC tissue and the correlation between LC3B and Cav-1 level in fibroblasts via QDs-based double immunofluorescence labeling. Our data demonstrated that high levels of fibroblastic LC3B correlate with lower invasion and possibly longer survival. Furthermore, fibroblastic Cav-1 and LC3B showed a significant positive correlation. Because LC3B is the essential component of the autophagosome membrane, a hallmark of autophagy, it is possible that autophagic degradation may not be responsible for the suppressed Cav-1 expression. However, these data are not consistent with the results in breast cancer, in which Lisanti and colleagues found that high oxidative stress is the root cause of autophagy in fibroblasts, leading to the autophagic degradation of Cav-1 [1,16,19,39]. Thus, a new puzzling question is raised: is the relationship between Cav-1 expression and autophagy in GC cooperative or antagonistic if Cav-1 is not degraded by autophagy? We found that low fibroblastic Cav-1 and negative LC3B expression represent trends of poor survival (*p* = 0.026 and 0.074, respectively), suggesting that Cav-1 and autophagy may act cooperatively in GC development. Indeed, identifying the role of autophagy in fibroblastic Cav-1 degradation or investigating the mechanisms that lead to Cav-1 degradation is a multistep task that involves more than analyzing its expression in tissues. More investigations are required to achieve these goals.

EBVaGC is defined by the presence of EBV in gastric cancer cells by EBER *in situ* hybridization and it constitutes approximately 8.7% of all GC cases, ranging from 2% to 18% [28,29]. In the current study, 17.79% (21/118) of GC cases were EBVaGC. This result is similar to the results published by others [28,29]. Because EBVaGC is a unique subtype of GC and it has features that are similar to the monoclonal proliferation of EBV-infected epithelial cells, it has several clinicopathological and molecular features, such as male predominance, low frequencies of intestinal phenotype mucins and low p53 expression [28,29]. Therefore, we tried to investigate whether EBV infection could impact the expression of stromal proteins in this study. Our result demonstrated that no difference in fibroblastic Cav-1 and LC3B expression was observed between EBVaGC and non-EBVaGC. Certainly, more work is needed to determine the influence of EBV infection on the tumor microenvironment, which may open a new avenue to treat EBVaGC patients.

Although the novel QDs-based multiplexed molecular imaging has been used in pathology-based research [40–42], it is not generally used in quantitative and semiquantitative studies. In our study, to ensure that our results were comparable with those of the other labs, we examined the status of Cav-1 and LC3B using the developed QDs-IHC method that was well-established and widely used in quantitative and semiquantitative studies [23,43–45] rather than using the developing QDs-based multiplexed molecular imaging technology. Furthermore, results from Yan Li and colleagues [40] and the current study showed that QDs-based multiplexed molecular imaging is highly suitable for observing the heterogeneity of cancer cells and stromal cells. However, the clinical uses of QDs-based multiplexed molecular imaging still need to be evaluated. Thus, conducting more studies to investigate the clinical significance of evaluating the heterogeneity of cancer or stromal cells via QDs-based multiplexed molecular imaging is recommended.

## 3. Experimental Section

### 3.1. Patients and Tissue Samples

A total of 123 formalin-fixed, paraffin-embedded (FFPE) GC specimens that were diagnosed from 2005 to 2008 were collected from the archives of the Department of Pathology, Zhongnan Hospital of Wuhan University, China. After the specimens were arrayed in TMAs, 118 (95.9%) cases contained adequate stroma were selected for further analysis. Among the 118 patients, 33 were female and 85 were male, with ages ranging from 24 to 82 years and an average age of 58 years. Based on the World Health Organization (WHO) histological criteria for GC, the specimen included 5 undifferentiated adenocarcinomas (ACs), 102 ACs and 11 mucinous ACs. For Lauren classification, 65 cases were intestinal-type, 8 were mixed-type and 45 were diffused-type GCs. According to the Union for International Cancer Control (UICC) TNM histology classification (2009), 24 were stage I, 29 were stage II, 62 were stage III and 3 were stage IV. Seventy-nine cases were lymph node metastasis-positive. The characteristics of the patient cohort are in agreement with the epidemiology and pathology of GC [14,46]. Two experienced pathologists (Yang, G.F. and Fan, L.F.) reconfirmed the histopathologic features of these samples. One hundred and seven patients were followed from the date of surgery until July 2012. The median follow-up time point was 62 months (range: 1–85). Overall survival was defined as the interval from the date of surgery to death. Patients who died of other diseases or due to unexpected events were excluded from this study. Written informed consent was signed by the patients, and approval for this study was obtained from the local Institutional Research Ethics Committee.

### 3.2. Tissue Microarray Construction

The H and E-stained sections of all specimens were reviewed, and the most representative tumor stromal areas were carefully selected for the construction of TMAs. The TMAs were constructed using a tissue-arraying instrument (Beecher Instruments, Silver Spring, MD, USA) as described in our previous study [44]. Briefly, two cores (diameter 1.5 mm) were punched from the selected area of each donor FFPE specimen and precisely arrayed in a recipient paraffin block. These blocks were consecutively cut into sections (4 μm thick) for making TMAs. Five sets of TMAs were constructed for Cav-1 and LC3B staining, Cav-1/LC3B double labelling, EBV detection and H and E staining.

### 3.3. QDs-Based Immunofluorescence Histochemistry

Cav-1 and LC3B expression was detected by QDs-IHC in two sets of TMAs. TMAs were deparaffinized in xylene and rehydrated in a graded ethanol series. Every step of QDs-IHC strictly followed the manufacturer’s instructions (Wuhan Jiayuan Quantum Dot Co., Ltd., Wuhan, China). Antigen retrieval was performed in citric acid (10 mM, pH 6.0) at 95 °C for 10 min, followed by cooling for 30 min. TMAs were first incubated in 2% BSA buffer at 37 °C for 30 min, and then at 4 °C overnight in rabbit anti-Cav-1 polyclonal antibody (diluted 1:300; Santa Cruz Biotechnology, Santa Cruz, CA, USA) and murine anti-LC3B monoclonal antibody (diluted 1:150; Cell Signaling Technology, Danvers, MA, USA) respectively, to permit antibody binding. TMAs were then washed three times with TBS-T (0.5% Tween, 0.1 M Tris-base, 0.9% NaCl, pH 7.6) for 5 min each time and incubated in biotinylated goat anti-rabbit or anti-murine IgG (1:100 dilution, Jackson ImmunoResearch, West Grove, PA, USA) at 37 °C for 30 min. For QD conjugation, antibody-binding TMAs were incubated in 2% BSA buffer again at 37 °C for 10 min, incubated in QDs (605 nm) and conjugated to streptavidin (QDs-SA) (1:200 dilution in 2% BSA, Wuhan Jiayuan Quantum Dot Co., Ltd., Wuhan, China) at 37 °C for 30 min, rinsed three times with TBS-T for 5 min each, and finally sealed with 90% glycerin (Sigma, St. Louis, MO, USA). The TMAs were observed under an Olympus BX51 fluorescence microscope equipped with an Olympus Micro DP 72 camera. The positive signal was bright red, target-specific and photo stable, and the background autofluorescence was green. Because Cav-1 is generally expressed in blood vessel endothelial cells, it was used as a positive internal control. Negative controls of Cav-1 and LC3B were carried out by replacing the primary antibodies with TBS.

The images of each specimen were independently evaluated by two experienced pathologists (Yang, G.F. and Fan, L.F.) who were blinded to the clinical features. H and E staining was conducted to identify the components of tumor tissue. The irregular ordered flat or fusiform cells with orbicular-ovate nucleolus in stroma compartment are termed fibroblasts in H and E staining. The sections were initially scanned at low power to select the most representative stromal areas for calculating. The samples containing no or only a few fibroblasts were eliminated from the analysis. We then counted the Cav-1- or LC3B-stained fibroblasts in five representative fields of each specimen at high magnification (200×) and estimated the positive area (PA) that was determined independently by the pathologists. The strong intensity dot-like or doughnut-shaped positive signal of Cav-1 in the tumor stroma was the internal control of endothelial expression. Scores of the two pathologists were compared and any discrepant scores were trained through reevaluating the staining by both pathologists to achieve a consensus score. PA was graded as follows: 0 (PA ≤ 20%), 1 (PA 21%–40%), 2 (PA 41%–60%) and 3 (PA > 60%). Then the intensity of staining (IS) was evaluated in hot spots at high-power magnification and was scored as: 0 (negative), 1 (weak) and 2 (strong). The stromal Cav-1 and LC3-B intensity distribution (ID) scores for each case were calculated by the following equation: ID = PA × IS, where ID ≤ 2 represented negative (−) or low and ID > 2 represented positive (+) or high. This standard was applied for Cav-1 and LC3B.

### 3.4. QDs-Based Double Immunofluorescence Labeling

We also detected the coexpression of Cav-1 and LC3B in GC tissue by QDs-based double immunofluorescence labeling. Every step followed the manufacturer’s instructions (Wuhan Jiayuan Quantum Dots Co., Ltd., Wuhan, China) and was the same as indicated in our previous study [45] with the following major steps: deparaffinizing→antigen retrieval→blocking→primary antibodies for Cav-1 and LC3B→washing→biotinylated goat anti-rabbit IgG (for Cav-1)→washing and blocking→605-QD-SA washing and QDs (545 nm) conjugated to goat anti-murine IgG (1:100, Wuhan Jiayuan Quantum Dots Co., Ltd, Wuhan China)→washing→mounting and observation. The QD fluorescent signal was analyzed using the Caliper multispectral microscopy imaging system (Caliper Life Sciences, Hopkinton, MA, USA). The Cav-1 positive signal was bright red and the LC3-B-positive signal was green. Negative controls were carried out in parallel, but the primary antibody was replaced with TBS, which had green autofluorescence. The double labeled serial sections were not formally scored but were used to visualize the relationship between fibroblastic Cav-1 and LC3B and to highlight the heterogeneity of tumor stroma.

### 3.5. QDs-Based Fluorescence in situ Hybridization

QDs-FISH was applied to detect the presence of EBER. The QDs-FISH kit, biotin-labeled EBER RNA probes (PanPath, B.V.: Amsterdam, The Netherlands), other reagents and routine steps were identical to our previous study in which a QDs-FISH imaging method of detecting EBV in GC was developed [47]. Briefly, TMAs were deparaffinized and hydrated, hybridized using biotin-labeled EBER RNA probes and incubated with QD-conjugated streptavidin. After rinsing, TMAs were detected using an Olympus BX51 fluorescence microscope equipped with an Olympus Micro DP 72 camera. The positive signal was red and the background autofluorescence was green.

### 3.6. Statistical Analysis

All data were analyzed by SPSS 17.0 software (SPSS Inc.: Chicago, IL, USA, 2008). Chi-square and Fisher’s exact tests were used to compare different rates. Correlations were calculated by the Spearman’s rank correlation test. The Kaplan-Meier method was used to evaluate the difference in overall survival. Univariate and multivariate analyses were conducted by the Log-rank test and Cox proportional hazard regression model. ROC curve analysis was used to determine the death predictive value of the parameters. Overall survival was defined as the interval from the date of surgery to GC-related death. Two-tailed *p*-values less than 0.05 were considered statistically significant.

## 4. Conclusions

This study revealed that low levels of fibroblastic Cav-1 can predict shorter survival time in GC, and high levels of fibroblastic LC3B correlate with less invasiveness and possibly a longer predicted period of survival. A combined Cav-1 and LC3B feature had a significant predictive value of death. Using the advanced QDs-based immunofluorescent imaging technology, we evaluated the coexpression of fibroblastic Cav-1 and LC3B in fibroblasts, making the positive correlation of Cav-1 and LC3B more vivid and easily comprehensible. As a unique subtype of GC, EBVaGC may not influence the fibroblastic Cav-1 and LC3B expression. Our results indicate the clinical importance of the stromal molecular expression of Cav-1 and LC3B and will potentially attract more attention to the exploration of the complicate tumor microenvironment.

## Figures and Tables

**Table 1:** Correlations between fibroblastic Cav-1/LC3B expression and clinicopathologic features of gastric cancer.

Features	*n*	Fibroblastic Cav-1		*p*	*n*	Fibroblastic LC3B		*p*
	
		Low (%)	High (%)			Negative (%)	Positive (%)	
Age				0.724				0.287
<58	55	28 (50.9)	27 (40.1)		50	39 (78.0)	11 (22.0)	
≥58	63	30 (47.6)	33 (52.4)		51	35 (68.6)	16 (31.4)	
Gender				0.186				0.384
Male	85	45 (52.9)	40 (47.1)		72	51 (70.8)	21 (29.2)	
Female	33	13 (39.4)	20 (60.6)		29	23 (79.3)	6 (20.7)	
Depth of invasion #				0.903				**0.032**
T1 + T2	36	18 (50.0)	18 (50.0)		32	19 (59.4)	13 (40.6)	
T3 + T4	82	40 (48.8)	42 (51.2)		69	55 (79.7)	14 (20.3)	
Lymph node status #				0.474				0.992
N0	39	21 (53.8)	18 (46.2)		30	22 (73.3)	8 (26.7)	
N1 + N2	79	37 (46.8)	42 (53.2)		71	52 (73.2)	19 (26.8)	
TNM stage #				0.725				0.914
0+Ia+Ib+II	53	27 (50.9)	26 (49.1)		44	32 (72.7)	12 (27.3)	
IIIa+ IIIb+ IV	65	31 (47.7)	34 (52.3)		57	42 (73.7)	15 (26.3)	
Grade of AC				0.792				0.065
Well and moderately	43	20 (46.5)	23 (53.5)		34	20 (58.8)	14 (41.2)	
Poorly	59	29 (49.2)	30 (50.8)		53	41 (77.4)	12 (22.6)	
Lauren classification				0.316				0.142
Intestinal-type	65	28 (43.1)	37 (56.9)		51	31 (60.8)	20 (39.2)	
Diffuse-type	45	26 (57.8)	19 (42.2)		42	36 (85.7)	6 (14.3)	
Mixed-type	8	4 (50.0)	4 (50.0)		8	7 (87.5)	1 (12.5)	
HER-2				0.967				0.628
Positive	23	11 (47.8)	12 (52.2)		23	16 (69.6)	7 (30.4)	
Negative	89	43 (48.3)	46 (51.7)		75	56 (74.7)	19 (25.3)	
Tumor cell type				0.565				0.189
AC	102	49 (48.0)	53 (52.0)		87	61 (70.1)	26 (29.9)	
MAC	11	7 (63.6)	4 (36.4)		10	9 (90.0)	1 (10.0)	
UC	5	2 (40.0)	3 (60.0)		4	4 (100.0)	0 (0)	
Fibroblastic Cav-1								**0.000**
Low					45	42 (93.3)	3 (6.7)	
High					51	28 (54.9)	23 (45.1)	

AC, Adenocarcinoma; MAC, Mucinous adenocarcinoma; UC, undifferentiated carcinoma;

# TNM classification of malignant tumors (7th edition).

*p* value in bold indicates *p* < 0.05.

**Table 2:** COX proportional hazard models of the overall survival of GC patients.

Factors	Univariate analysis		Multivariate analysis	

	*p value*	HR (95%CI)	*p value*	HR (95%CI)
Sex				
Men *vs*. women	0.732	1.098 (0.638, 1.890)		
Age				
<56 *vs*. ≥56	0.199	0.728 (0.445, 1.193)		
TNM stage				
I–II *vs*. III–IV	**0.003**	0.543 (0.256, 0.803)	**0.001**	4.344 (1.895, 9.958)
T classification				
T1-T2 *vs*. T3-T4	**0.001**	0.317 (0.137, 0.732)	0.483	
Lymph node				
No *vs*. Yes	**0.034**	0.528 (0.275, 0.987)	0.663	
Lauren classification				
Intestinal-type *vs*. Diffuse-type	0.882	0.965 (0.603, 1.543)		
Grade of AC				
Well and moderately *vs.* poorly	0.638	0.873 (0.493, 1.546)		
Fibroblastic Cav-1				
High *vs*. Low	**0.021**	0.556 (0.332, 0.929)	**0.029**	0.474 (0.242, 0.928)
Fibroblastic LC3B				
Positive *vs*. Negative	**0.041**	0.498 (0.235, 1.055)	0.180	
EBV infection				
Yes *vs*. No	0.347	0.725 (0.355, 1.481)		

*p* value in bold indicates *p* < 0.05.

**Table 3:** The relationship among EBV infection, fibroblastic Cav-1 and LC3B in GC tissues.

Variables		Fibroblastic Cav-1				Fibroblastic LC3B			
	
		Low (%)	High (%)	*r*	*p*	Negative (%)	Positive (%)	*r*	*p*
		
EBV Infection	Negative	48 (49.5)	49 (50.0)	0.092	0.877	60(74.1)	21 (25.9)	0.037	0.712
	Positive	10 (47.6)	11 (52.4)			14(70.0)	6 (30.0)		
